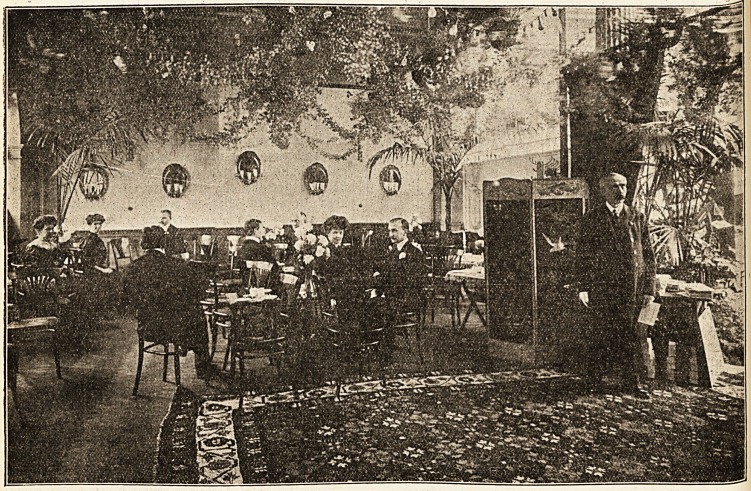# The Medical Exhibition

**Published:** 1909-10-09

**Authors:** 


### The Medical Exhibition.

### AN INTERESTING EVENT OF THE YEAR.

The fifth medical exhibition organised by the "British and Colonial Druggist" opened on Monday last, in the Horticultural Hall, Vincent Square. In point of interest, in excellence of display and originality of arrangement, it yields to none of its predecessors, excellent as they have been; and the practitioner who wishes to spend an hour or two in an instructive fashion cannot do better than follow the example of the many hundreds of medical men who, with their friends, have been wandering round the exhibition in pleasure cruises among the novelties. There is enough and to spare for him who is eager to meet new drugs, appliances, or things medical in any line or department. The arrangements for the visitors' comfort have been much appreciated by all who have been to see the exhibition, and the thanks of practitioners and their friends are due to Mr. Flack, the courteous General Secretary, and his staff of assistants, who did their utmost to welcome the visitor and make his visit an enjoyable one.

### The Exhibits.

Where so much that is excellent claims attention, it is not an easy task to single out any of the eighty odd stalls for special comment. Nevertheless, it must be attempted, and a walk round the hall is a very helpful aid to the selection. At Nos. 1 and 2 the London Warming and Ventilating Company, Limited, exhibit their stoves, while No. 3 is the attractive booth of Brand and Company, makers of the world-famous specialties for invalids. Here one finds home-made beef-tea, chicken, beef, turtle, and other jellies, beef peptone, and a huge pyramid of meat extract to show the company's latest "solid extract," a production which is well worth sampling. Next to it is Chesterman and Streeter's stall, with an exhibit of rubber goods, flanked by the exhibit of the Seamless Rubber Company. Then comes W. B. Saunders Company, medical

Army Medical Library Washington, D. C. U. S. A.

publishers, with the Liebig Extract of Meat Company's stall alongside it. A fine display this latter, with Lemco and Oxo, and the delicious Fray Beutos ox tongues and ox-tail soups. A feature is Bifti, the pure penny beef tablet. Horlick's Malted Milk is found at Stands 8 and 9; at 10 there is another medical publisher, this stall housing the fine books for the publishing of which the J. B. Lippincott Company is responsible. Stand 11 is a monument to the energy and variety of Messrs. Cadbury, and here the visitor may sample a cup of that most exquisite of cocoas, the Bourneville brand. At Stand 12, tenanted by the Hygienic Bath Company, are found the Zana Effervescing Baths, which are a novelty that should interest visitors. Next to this stall the Hoffmann la Roche Chemical Works show attractive specimens of their fine products, such as digalen, thiocol, seccacomin, airol, and thigenol. Then come Mrs. Cameron, who shows various forms of corsets; volumes of Mr. Burghard's "System of Surgery" to the little manuals for the practising physician. They represent the perfection of modern medical publications. Callard and Company show interesting new diabetic breads; Welford and Sons show all kinds of milk and milk foods; while in the same row we come across Hall's Wine, Australian Burgundy and Carvino, the only true Lemco wine, all exhibited by Stephen Smith and Company. Then comes a most attractive and interesting stall, that of Duncan, Flockhart and Company; of the many novelties it contains we can only mention the excellent preparations containing formates, as these are not sufficiently well known. The capsule formate co. and fluid extract of malt with formate (Duncan) are preparations which contain the double formates and which well merit an extended trial. Gautier Frères show their fine cognac brandies and equally fine Angelus liqueurs at Stand 31; at 32 are the various

Ernst Leitz, of microscope fame; the Manhu Food Company, with a fine display of diabetic foods; George Back and Company with a display of diabetic whisky; Hanneman and Son, whose biscuits every visitor is invited to nibble; Bruce Green and Company, Limited, opticians; and the Grape Juice Company, showing Mostelle, the pasteurised unfermented juice of fresh sun-ripened Spanish grapes, a pure grape juice that is equal to the finest Hallauer. At 22 is the temporary home of Regulin, prepared by the Helfenberg Chemical Works, an agar-agar compound which has been extensively used in cases of chronic constipation. Stand 23 is noteworthy because here the visitor sees Lee's Antiseptic Air Producer, which is now used in many hospitals and sanatoria, and which appears to us to be as superior to the old bronchitis kettle as a modern Leitz lens is to the glasses that Spinoza polished. The Oxford Medical Publications are massed together at Stand 25, and a fine show they make, too, from the grand specialities of Messrs Kirby and Company. Then come John Bell and Croyden, with dressings, model sterilising-rooms, and the Droitwich Brine Crystals; the Plasmon stall, the Aylesbury Dairy stall, where one can taste kephir, koumiss, and other weird lactine preparations; the Fairchild Bros. and Foster stall, with pepsin compounds, and the Caledonia Springs Company stall, where magi water is on draught. At Stalls 40 and 41 the Hewlett preparations are shown, with a fine selection of instruments and apparatus; at 42 the medicated wines of Beaufoy and Company; and at 43 the many special novelties manufactured by the Bayer Company. Ingram and Royle show all known natural waters, salts and pastilles, and next to them is the Park Davis stand with innumerable preparations. Then we come across a very old and tried friend, Angier's Emulsion of petroleum with hypophosphites, prepared by the Angier Chemical Company. Schweppe's, at Stand 55, show a new pure fruitjuice, Vindevie, which is of excellent flavour and quality; while the Jeyes' Sanitary Compounds Company has an attractive stall. At 58 the A. and M. Zimmermann stand, there is a fine selection of Continental drugs and sera; while further on Merck, Oppenheimer, and the Saccharin Corporation have equally interesting displays of specialties. The Medical Supply Association shows a great variety of surgical instruments, nursing requisites, and hospital furniture; the Greville operating-tables being a special feature of this display. Then we renew acquaintance with drugs and specialties we already know well; at the stands of Messrs. Corbyn and Stacey, Thomas Christy and Co., Antiphlogistine, McClintons, Wander (Ovaltine), Wincarnis (Coleman and Company), Stern, Australian Wine Company, the Hanley Chemical Manufacturing Company, Casein Limited (whey powder), Friedrichshall water, Johnson and Johnson (surgical dressings), Salutaris Water Company, Hawksley and Son (surgical instruments), Maltine, Clay, Paget and Company (infant foods), Anglo-American Pharmaceutical Company, Keen Robinson and Company, of mustard fame, Wooley, of Manchester, Martindale, whose stall is indeed worth half an hour's inspection, Virol, Limited, Bovril, Limited, Lifebelt Coffee, Fassett and Johnson (showing Seabury products), Findlater and Company, Newton, Chambers and Company (Izal products), Arthur Cox and Diamalt.

### Some Special Features.

A few stalls we lingered at longer than others, perhaps invidiously, but they seemed to us to merit more than others the attention of the general practitioner. Here, for instance, at Stand 62, was Mr. C. A. Hoefftcke himself, representing his firm (Oxford and Bond Streets) of orthopædic apparatus makers. Every variety of apparatus and appliance is here, or if it isn't Mr. Hoefftcke, a cheery enthusiastic Netherlander, who is a prince of fitters, will make it for you. The modified Hessing apparatus shown here is well worth noticing, especially as it is carefully demonstrated and X-ray photographs of actual cases shown with it.

The Motosacoche, Ltd., show models of their 1910 motor-cycle, eminently suitable for professional men. This is an ideal motor-cycle, which can run 140 miles on a mere gallon of petrol. Moreover, Motosacoche, even if you can't pronounce the name, will allow you to try the machine free, which, as the notices say, is really better than talking about its merits.

Mr. James Barker shows at Stand 79 his Barker Vibrators, which are quite surprisingly smooth running, and in advance of most models on the market. At 89 are to be found dozens of bottles of the *Rocla* Natural Tonic Co. Rocla is an Italian Government mineral water, the only known natural mineral tonic. It is a very old preparation obtained by filtration through manganese rock and is very rich in iron. It appears to us a very good preparation to use in cases where inorganic iron is indicated.

The Hospitals and General Contracts Co. has a most interesting and up-to-date display, which gives a good idea of the variety of things to be seen at the show-rooms at 33 and 35 Mortimer Street. The visitor is easily impressed by the Porcellite enamel hospital furniture, which does not chip, change colour or fade, and which is practically indestructible. It is now to be seen at such institutions as the London and Middlesex Hospitals and the Manchester Infirmary. This firm shows a variety of instruments which are excellent and inexpensive, and, indeed, it appears that excellence and cheapness are features of everything which figures in the display.

Bratt, Colbran and Co., of 10 Mortimer Street, show a special exhibit of their heaped fire and gas fire, which interested us much. These are really most economical and simple contrivances, and are now being put in the wards of most of the metropolitan institutions. For simplicity, ease of working, and freedom from smell or smoke, the practitioner who desires to heat his consulting room or study cannot do better than invest in a "heaped gas fire."

At Stand 80 visitors were mouthing morsels of Bipsine, a white digestive bread, which is bound to become popular; and at 110, Taunton's, Ltd., show a choice variety of their institutional appliances which hospital secretaries and superintendents would do well to inspect. Their Dublin Rotunda and Brighton surgical bedsteads are models of what such things should be.

## Figures and Tables

**Figure f1:**